# Quality of Life after Motorcycle Traffic Injuries: A Cohort Study in Northwest of Iran

**DOI:** 10.30476/BEAT.2021.87236.1182

**Published:** 2021-10

**Authors:** Leili Abedi Gheslaghi, Hamid Sharifi, Mehdi Noroozi, Mohsen Barouni, Homayoun Sadeghi-Bazargani

**Affiliations:** 1 *Student of Epidemiology, Department of Biostatistics and Epidemiology, School of Public Health, Kerman University of Medical Sciences, Kerman, Iran*; 2 *HIV/STI Surveillance Research Center, and WHO Collaborating for HIV Surveillance, Institute for Futures Studies in Health, Kerman University of Medical Sciences, Kerman, Iran*; 3 *Psychosis Research Center, University of Social Welfare and Rehabilitation Sciences, Tehran, Iran*; 4 *Health Services Management Research Center, Institute for Futures Studies in Health, Kerman University of Medical Sciences, Kerman, Iran*; 5 *Road Traffic Injury Research Center, Tabriz University of Medical Sciences, Tabriz, Iran*

**Keywords:** Quality of life, QOL, Motorcycle, Traffic accident, Cohort study, Hospitalization, Patient discharge, Iran

## Abstract

**Objective:**

To investigate the quality of life (QOL) of injured motorcyclists and associated factors in a period of three months after the accident**.**

**Methods:**

In the present study, we were included 190 injured motorcyclists who admitted to two referral specialized hospitals (Emam Reza and Shohada) in Tabriz, between June 2018 and January 2019. All injured motorcyclists were contacted through the telephone one and 171 of them (90%) three months after their accident to complete an EQ-5D-3L questionnaire. The baseline measurements were gathered by using face to face interviews in the hospitals. The QOL score could vary between 1 and 3. The higher score showed a lower QOL.

**Results:**

The injured motorcyclist’s QOL score was relatively better in three months after the accident (mean±Standard Deviation (SD): 1.78±0.51) in comparison with their status a month after the accident (2.15±0.65) (*p*<0.001). The multivariable model showed that individuals with pelvis injuries (Coef: 0.29, (95% CI: 0.16, 0.42), *p*=0.001) and knee injuries (Coef: 0.26, (95% CI: 0.10, 0.42), *p*=0.001), experienced a higher QOL score. Also, those whose accident had happened in rainy weather experienced higher QOL score (Coef: 0.33, (95% CI: 0.12, 0.53), *p*=0.001). The patients who were in an accident with a vehicle were experienced a better QOL than others (Coef: -0.26, (95% CI: -0.43, -0.09), *p*=0.002).

**Conclusion:**

The assessment of three-months post-accident showed that the QOL score of the motorcyclists was reduced. It is recommended that the QOL of patients should be improved in hospital discharge victims.

## Introduction

According to the World Health Organization’s (WHO) report, 1.35 million individuals are killed due to road traffic injuries (RTIs) and more than 50 million are injured or disabled every year [1, 2]. Among the road accidents, those related to motorcycles are specifically important and impose a high burden on low and middle income countries, such as Iran, compared to the high-income ones [3]. Based on the WHO’s report, in Iran, 2016, the rate of death from RTIs was 20.5 per 100,000 peoples, 24% of which belonged to motorcycle riders [2]. Thus, premature death and disability with long-term consequences impose a considerable burden on people and society [4]. The effects of non-fatal injuries include both the physical aspect of RTIs and the psychosocial factors following the injury. The physical consequences of injuries have received much more academic attention, while the psychological aspects have been neglected [5]. Therefore, the quality of life (QOL) of the survivors from these accidents is a critical issue to be assessed and addressed [6].

Studies conducted on QOL have shown this concept which has a multidimensional structure including physical, psychological, and social [7]. According to WHO’s guidelines, QOL is a subjective and dynamic concept determined by the affected people, not by any other person; it changes dynamically over time and, thus, should be measured within a specified period [8].

A considerable volume of literature exists on the QOL of the RTIs’ victims [9-14]. The results of a prospective cohort study in France assess the QOL of the RTIs victims through one-year follow-up that showed head injury, severe injury, intention to complain, and early post-accident medical complications were predictive of health dissatisfaction. Moreover, post-traumatic stress disorder (PTSD) and socio-economic problems are believed to be associated with poor QOL [15]. A prospective cohort study in Germany were assessed the health-related QOL two years after a traumatic experience of severely injured patients. They demonstrated that more than 60% of them showed relevant persistent pain and severe functional deficit in at least one body region [16]. Moreover, several longitudinal cohort studies have shown that individuals who suffer from injuries following RTIs report long-term life consequences (such as physical, psychological, financial, and everyday life consequences) and there are psychological reactions like travel anxiety and symptoms like PTSD, which have been reported more by women. Therefore, women compared to men have poorer QOL. Moreover, psychological reactions such as PTSD and QOL have been reported in victims after RTIs [17-19].

In comparison to other vehicle users, motorcyclists suffer from more severe and multiple injuries especially to the arm and adjacent area. Most of the motorcyclists have a poor health status about three years after the accident. Also, the rate of anxiety and fear of traveling is the highest among riders and passengers [20]. In accidents, the QOL of injured motorcyclists is conducted much less especially in developing area and in the context of Iran. Therefore, this study aims to investigate the QOL of injured motorcyclists and the associated factors in a period of three months after the accident.

## Materials and Methods

The present study is a part of the Persian Traffic Cohort (PTC) study and the cases were selected from Iranian Integrated Road Traffic Injury Registry (IRTIR) system. It should be noted that the Ministry of Health and Medical Education designed IRTIR with the collaboration of WHO. The comprehensive IRTIR system has been established in Emam Reza and Shohada hospitals as the two referral specialty centers in East Azarbaijan Province, Iran. The IRTIR gathers data at several sections as follows: crash scene, emergency, hospital admission, forensic medicine, and post-discharge. 

The sample size for this study was estimated based on a pilot study. The pilot study was conducted on 30 injured persons. The results were used to calculate the sample size. The following formula was utilized to calculate the sample size with standard deviation of σ = 0.7, accuracy of d = 0.10, and α = 0.05 for 196 participants.

$n=\frac{Z_{1-\frac{\alpha }{2}}^{2}\delta^{2}}{d^{2}}$

We recruited 196 injured motorcyclists admitted to the two referral specialty hospitals of Emam Reza and Shohada in Tabriz from June 2018 to January 2019. Among them, we could contact 171 injured people three months after the accident. Out of 196 patients, 6 had no corporation with researchers. The inclusion criteria were: 

1) Being involved in traffic injury (according to United Nations Economic Commission for Europe’s (UNECE) definition [21]”, road traffic accidents are those accidents:

a) Which occur or originate on a way or street open to public traffic;

b) Which result in one or more persons being killed or injured;

c) In which at least one moving vehicle is involved.”

2) Being a rider or pillion passenger of motorcycle involved in traffic crash in accordance with V20-V29 and V31 from International Classification of Diseases-10^th^ revision (ICD-10);

3) Having registered integrated road traffic injuries (hospitalization in trauma centers);

4) Having the participant’s consent for inclusion in the study;

5) Being lucid, conscious, and cooperative during the telephone follow-up.

Those injured individuals who either were in a coma during the phone follow-up or could not talk due to severe pain were excluded.

*Data Collection*

For each injured motorcyclist (both the rider and the passenger ‘if applicable’) admitted to the hospitals (Emam Reza and Shohada), the baseline measurements were collected through a face-to-face interview in the hospital admission section of IRTIR through data collection tool in the nursing station. 

*Baseline Measurements*

The baseline data were gathered using a face-to-face interview at the hospitals:

1) Demographic characteristics (age, sex, level of education, marital status, job, length of stay in hospital).

2) Crash-related variables including:  

a) Data regarding the time and location of the accident including the following items:

The day, month, and year of the accident; 

The exact day of the week;

Time of accident occurrence (hour);

Accident time light status (including daytime, nighttime);

 Weather conditions during accident (including sunny, cloudy, rainy);

Road condition at the time of the accident, and whether it was slippery or not;

Occurrence of accident inside or outside the city.

b) Data regarding the vehicle including the following items:

The number of vehicles involved in the accident (including single vehicle, multiple vehicles);

The type of vehicle engaged in the accident (including no vehicle, bicycle, motorcycle, car, bus, and heavy car);

The mechanism of accident (including vehicle-fixed object accident, vehicle-vehicle, overturning, vehicle-pedestrian, and vehicle-animal accident).

c) Information about the person including the following items:

The role of the injured person (including whether s/he is the rider or the passenger);

Having riding license if the injured person is the rider;

Riding history of the rider (including less than 1 year, 1-3 years, 3-10 years, and more than 10 years);

Average riding hours per day during the last week in the rider (including less than 1 hour, 1-4 hours, 4-7 hours, and more than 7 hours);

Helmet use during accident

Rider’s conversation with passenger before accident occurrence (including without passenger, no conversation with passenger, routine conversation with passenger, and controversy with passenger);

Number of riding days during last week.

All these data were recorded in the comprehensive IRTIR system.

3) Data regarding the accident injuries severity was extracted from health information system (HIS) of the hospitals. The type of injuries was defined according to ICD-10 codes as follows: head injuries (S00-S09), neck injuries (S10-S19), thoracic injuries (S20-S29), injuries to the abdomen, lower back, lumbar spine, and pelvis (S30-S39), shoulder and above elbow injuries (S40-S49), elbow and forearm injuries (S50-S59), wrist and hand injuries (S60-S69), leg and pelvis injuries (S70-S79), knee and lower knee injuries (S80-S89), and foot and ankle injuries (S90-S99).

*Follow-up Assessment Including QOL*

One and three months after the accidents, the injured hospitalized motorcyclists were contacted by telephone for a follow-up. Required information for performing the follow-up was collected from the IRTIR system. The call duration for each person lasted from five to ten minutes.

Questionnaires

In this study, we used two questionnaires to collect the necessary data:

A) The EQ-5D-3L questionnaire was developed in 1987 by a team of researchers from five European countries to assess the QOL. This standard questionnaire covers five aspects of mobility, self-care, usual activities, pain/discomfort, and anxiety/depression. For each of these aspects, the following scale is considered: 1) I have no problem; 2) I have some difficulty; and 3) I have many issues. The overall score is calculated as the sum of the scores obtained from each aspect divided by 5. The average overall score is between 1 (high QOL) and 3 (low QOL) [22]. A higher score shows a lower QOL. The Persian format of the EQ-5D-3L questionnaire was first used in this study. The questionnaire was validated before conducting the study. 

B) The Iranian short version of socio-economic status assessment questionnaire (SES-Iran) covers six items includes occupation, income per month, years of successful education, private housing, private car, and share of health expenditure to total expenditure. To classify the socio-economic status to very low, low, medium, and high levels, statistical quartiles of the overall score were used. The validity and reliability of this questionnaire were proved by Dr. Sadeghi *et al*., [23].

Statistical Analyses

Descriptive statistics for normal quantitative has been reported as mean (standard deviation (SD), for not normal quantitative; it is as median (P25-P75), and for qualitative variables, it is as frequency (percent), respectively. We checked the normality of continuous variables (including age, number of riding days during last week, time of accident occurrence, hospital admission days, and quality of life score) by the Shapiro-Wilk test. Demographic variables, baseline variables, and QOL score were compared in the first and second follow-up using Chi-square test, Mann Whitney, and independent t-test.

In the present study, our data were longitudinal and the generalized estimating equations (GEE) model was undertaken to analyze change in outcomes over time and to explore predictors of these outcomes. Therefore, bivariable and multivariable linear models of the GEE with unstructured variance-covariance matrix were used to determine the factors affecting the QOL of injured motorcyclists. In this model, the QOL score was the dependent variable and the baseline variables were considered as independent. 

In the bivariable model, we entered all the demographic variables (included age, sex, level of education, marital status, and job) and baseline variables into the model. Therefore, we tested the association of these variables with the QOL score. In the multivariable GEE model, the variables with *p*-value <0.1 [24] in the bivariable were included in the model. In these models, a positive coefficient showed lower QOL and a negative coefficient showed better QOL. Also, magnitude of the coefficients meant a decrease in the QOL of the patients. The backward elimination by using Wald test was used to reduce the model. In all analyses, *p*<0.05 was considered statistically significant. Stata SE software (version 13) was used for the data analysis. 

Ethical Considerations

This study was approved by Ethics Committee of Kerman University of Medical Sciences (ethics code: IR.KMU.REC.1397.141) and carried out under the national ethical codes for the primary cohort and registry. Also, verbal consent was received from all the participants before enrollment.

## Results

Basic Measurement

Totally, 190 injured patients in 250 accidents were included in the study. The mean±SD age of the participants was 29.65±14.02; more than sixty percent of them had an education level higher than six classes. Five (2.6%) injured participants were women and 103 (53.7%) were single. Moreover, 160 (85%) of the injured participants were riders and only 37 (19.8%) individuals were wearing helmets at the time of the accident. Almost 53 (32.9%) of the injured motorcyclists had over ten years of riding experience and rode the motorcycles for one to four hours during the week. Two-thirds of injured riders did not have a rider’s license. Totally, 173 (92.0%) accidents occurred on sunny days and only 11 (5.8%) roads were slippery. Most accidents 97 (90.9%) occurred on the main roads in the city (Table 1).

**Table 1:** Demographic and Injury Characteristics of Motorcycle Riders Involving a Motorcycle accident at 1 Months and3 Months Post injury, Iran, in 2018

**Variables**					**After 1 month follow- up (n=190)**	**After 3 months follow- up (n=171)**	***p*** **-value**
Individual information							
Quality of life score; Mean (SD)					2.15 (0.6)	1.78 (0.5)	<0.001^ b^
Age (years); Median(P25-P75)					26 (20-35.5)	26 (20-35)	0.54^c^
Education level n (%)		Illiterate or up to 6 classes			75 (39.9)	65 (38.5)	0.91^ a^
		Higher than 6 classes			113 (60.1)	104 (61.5)	
Sex n (%)		Male			185 (97.3)	164 (97.1)	0.87^ a^
		Female			5 (2.6)	5 (2.9)	
Marital status n (%)		Single			103 (53.7)	92 (54.4)	0.76^ a^
		Married			88 (46.3)	77 (45.6)	
Job of injured persons n (%)		Employee			5 (2.6)	3 (1.8)	0.99^ a^
		Farmer & rancher& Worker			70 (36.8)	61 (36.1)	
		Artesian			19 (10)	19 (11.2)	
		Others			81 (42.6)	73 (43.2)	
		Without job			18 (7.9)	13 (7.8)	
Role of injured persons n (%)		Rider			160 (85.1)	147 (86.9)	0.69^ a^
		Passenger			28 (14.9)	22 (13.02)	
Having riding license if the injured person was rider n (%)		Yes			26(16.2)	25 (17)	0.98^ a^
		No			126 (78.7)	115 (78.2)	
		Unknown			8 (5)	7 (4.7)	
Riding history of rider n (%)		Lower 1 year			10 (6.2)	8 (5.5)	0.98^a^
		1-3 years			27 (16.7)	21 (14.4)	
		3-10 years			66 (40.9)	59 (40.4)	
		Over 10 years			53 (32.9)	53 (36.9)	
		Unknown			5 (3.1)	4 (2.7)	
Average riding hours per day during the last week in rider n (%)		Lower 1 hour			31 (19.3)	26 (17.8)	0.9^a^
		1-4 hours			69 (42.9)	67 (45.9)	
		4-7 hours			38 (23.6)	33 (22.6)	
		Over 7 hours			11 (6.8)	9 (6.2)	
Helmet use n (%)		Yes			37 (19.8)	34 (20.2)	0.98^a^
		No			145 (77.5)	127 (75.6)	
Rider’s conversation with passenger before accident occur; n (%)		Without passenger			3 (2.2)	3 (2.6)	0.98^ a^
		Not convers with passenger			97 (71.8)	86 (73.5)	
		Routine convers with passenger			32 (23.7)	26 (22.2)	
		Controversy with passenger			3 (2.2)	2 (1.7)	
Number of riding days during last week; Median(P25-P75)					6.5 (4-7)	7 (4-7)	0.58^c^
Socio –economic status n (%)		Quartile 1 (Very low)			41 (25.7)	-	
		Quartile 2 (low)			41 (25.7)	-	
		Quartile 3 (Medium)			37 (23.3)	-	
		Quartile 4 (High)			40 (25.2)	-	
Hospital admissions days				Median(P25-P75)	3 (2-5)	3 (3-5)	0.66^c^
Time and location of accident							
Day of accident n (%)		Saturday			23 (12.2)	18 (10.5)	0.99^ a^
		Sunday			32 (17.02)	28 (16.4)	
		Monday			18 (9.6)	18 (10.5)	
		Tuesday			29 (15.4)	28 (16.4)	
		Wednesday			24 (12.8)	24 (14.04)	
		Thursday			29 (15.4)	26 (15.2)	
		Friday			33 (17.5)	29 (16.9)	
Occurring of accident in holiday; n (%)		Yes			34 (18.1)	27 (15.5)	0.76^ a^
		No			154 (81.9)	144 (84.2)	
Time of accident occur (hour)			Median (P25 to P75)		13 (9 to 20)	17 (13 to 20)	0.48^c^
Lighting intensity during accident; n (%)		Day			126 (67.02)	112 (66.3)	0.97^a^
		Night			57 (30.3)	53 (31.4)	
Weather condition during accident; n (%)	Sunny				173 (92.02)	159 (92.9)	0.78^a^
	Cloudy				8(4.3)	5 (2.9)	
	Rainy				7 (3.7)	7 (4.1)	
Slippery condition during accident; n (%)	Dry				174 (92.5)	159 (92.9)	0.96^ a^
	Slippery				11 (5.8)	9 (5.3)	
Occur of accident in residential zone; n (%)	Yes				119 (63.3)	104 (60.8)	0.96^ a^
	No				68 (36.2)	66 (38.6)	
Intercity accidents; n (%)	Main road				97 (90.9)	86 (89.6)	0.83^ a^
	Byroad				10 (9.3)	10 (10.4)	
Outer city accidents; n (%)	Main road				21 (25.9)	22 (29.3)	0.97^ a^
	Byroad				59 (72.8)	52 (69.3)	
Vehicle information							
Number of vehicle involved in accidents; n (%)	Single vehicle				44 (23.4)	43 (25.2)	0.88^ a^
	Multivehicle				141 (75)	126 (73.7)	
Type of vehicle engage; n (%)	Don’t have vehicle				44 (23.4)	42 (24.6)	0.96^ a^
	Others (Bicycle, Motorcycle, bus, heavy car)				32 (17.02)	26 (15.2)	
	Car				112 (59.6)	103 (60.2)	
Mechanism of accidents; n (%)	Vehicle-Fixed object				11 (5.8)	10 (5.8)	0.99^ a^
	Vehicle-vehicle				139 (73.9)	124 (72.5)	
	Overturning				23 (12.2)	23 (13.4)	
	Others(vehicle-pedestrian, vehicle-animal, falling, Exit from the road)				11 (5.8)	11(6.4)	
Type of injuries							
Head injury; n (%)	Yes				52 (27.7)	41 (23.9)	0.57^ a^
	No				136 (72.3)	130 (76.02)	
Thorax injury; n (%)	Yes				12 (6.4)	11 (6.4)	0.98^ a^
	No				176 (93.6)	160 (93.6)	
Neck injury; n (%)	Yes				4 (2.1)	4 (2.3)	0.82^ a^
	No				184 (97.9)	167 (97.6)	
Shoulder and upper arm injuries; n (%)	Yes				18 (9.6)	18 (11.1)	0.4^ a^
	No				170 (90.4)	153 (88.9)	
Elbow and forearm injuries; n (%)	Yes				19 (10.1)	19 (11.1)	0.75^ a^
	No				169 (89.9)	152 (88.9)	
Wrist and hand injuries; n (%)	Yes				11 (5.8)	11 (6.4)	0.38^ a^
	No				177 (94.1)	157 (93.6)	
Hip and thigh injuries; n (%)	Yes				27 (14.4)	26 (15.2)	0.82^ a^
	No				161 (85.6)	145 (84.8)	
Knee and lower leg injuries; n (%)	Yes				69 (36.7)	59 (34.5)	0.66^ a^
	No				119 (63.3)	112 (65.5)	
Ankle and foot injuries; n (%)	Yes				15 (7.9)	12 (7.02)	0.73^ a^
	No				173 (92.02)	159 (92.9)	
Abdomen & lower back & lumbar & spine & pelvis injuries; n (%)	Yes				20 (10.6)	18 (10.5)	0.97^ a^
	No				168 (89.4)	153 (89.5)	

^a^ *p*-value based on chi-square test; ^b^ P-value based on t independent test; ^c^ *p*-value based on Mann–Whitney test

QOL Score

The mean±SD of QOL score injured motorcyclists in the first month after the accident was 2.15±0.65 and it was 1.78±0.51 in the three months after the accident. The QOL score within the third month of their follow-up was lower than the first month after the accident. It means that the QOL score of the participants improved in the second follow-up (*p*<0.001) (Table 1). 

Predictors of QOL Score

Bivariable analysis showed that being in rainy days (Coef: 0.45, *p*=0.001), no vehicle involved (Coef: -0.42, *p*=0.001), motorcycle overturn (Coef: -0.54, *p*=0.001), one vehicle involved in the accident (Coef: -0.39, *p*=0.001), being the passenger (Coef: -0.13, *p*=0.03), knee injuries (Coef: 0.34, *p*=0.001), pelvis injuries (Coef: 0.30, *p*=0.001), and role of the injured person (Coef: 0.27, *p*=0.003) affected QOL of the injured people in motorcycle accidents.

In the final model, we had five variables includes role of the injured person, pelvis injuries, knee injuries, number of vehicles involved in the accident, and weather conditions during accidents. Based on the final model, the individuals with pelvis injuries (Coef: 0.26, *p*=0.001), and knee injuries (Coef: 0.29, *p*=0.0001) had experienced higher QOL score than others. Accidents that had occurred on rainy days, in comparison to those that happened on sunny days were increased the QOL score of the injured individuals (Coef: 0.33, *p*=0.001). Those who were in an accident with a vehicle were experienced a lower QOL score than others (Coef: -0.26, *p*=0.002) (Table 2).

**Table 2:** Multivariable Generalized Estimating Equations (GEE) assessing the change of a sample of hospitalized motorcycle riders involving a motorcycle accident in Iran, in 2018

**variables**		**Multivariable GEE**		
		**B**	**95% Confidence. Interval**	***p*** **-value**
Role of injured persons	Rider	Ref	Ref	Ref
	Passenger	-0.14	(-0.31 , 0.03)	0.11
Hip and thigh injuries	Yes	0.26	(0.10 , 0.42)	0.001
	No	Ref	Ref	Ref
Knee and lower leg injuries	Yes	0.29	(0.16 , 0.42)	0.001
	No	Ref	Ref	Ref
Number of vehicles involved in accident	Single vehicle	-0.26	(-0.43 , -0.09)	0.04
	Multivehicle	Ref	Ref	Ref
Weather condition during accident	No rainy	Ref	Ref	Ref
	Rainy	0.33	(0.12 , 0.53)	0.001

## Discussion

The results of the present study showed that the QOL score of the injured motorcyclists was high a month after the accident, which was consistent with the results of a similar study conducted in India [24]. Comparing the mean score of the QOL in this study to that of the general population in Australia [25], China [26], and Iran [27] showed that the QOL scores of the general population in Australia, China, and Iran were 0.9, 0.98, and 0.74, respectively, which were significantly higher than the QOL score of the injured after one month of follow-up.

Our study showed that QOL score of injured people in motorcycle accidents, three months after the accident, was partially improved when compared to the QOL score one-month post-accident. This finding is consistent with the results of a study in Australia [14]. The results of a systematic review also showed an increase rate in an injured people’s QOL immediately after the accident [19]. In comparison to other injured individuals, a study specifically examined the QOL score of injured motorcyclists over three months after their accident and showed that they continued to require medical care and suffered from severe injuries from three months to a year after their accident [20]. Hence, more studies are required on injured motorcyclists to help understand whether their QOL score improvement is due to their adjustment to the aftermath of the accident or the reduction of their disabilities and improvement of their symptoms. 

Based on the results of a review [19], the factors affecting the QOL score of traffic accident victims are categorized into four groups: 1) demographic variable, 2) clinical variables, 3) psychosocial variables, and 4) socioeconomic variables. Regarding the demographic variables, there was no significant relationship between age or sex and the QOL score of the injured participants in the present study. This is consistent with the results of other studies [12, 28]. Yet, our results are inconsistent with the results of cohort studies conducted in France and Australia where injured adults (usually above 35 years old) have had a poorer QOL than the youth [15, 29]; comparison to men, women have had a poorer QOL [9]. Accordingly, from two to eight months after their accident, women tended to return to their job 18% less than men due to the persistence of their medical issues [30]. One of the reasons for the inconsistency between these two studies is the difference in the pattern of RTIs, since in both Australian and French studies, all RTIs were studied, but the present study focused on motorcycle accidents. Moreover, the number of women included in this study was very low and this could not consider the association between gender and QOL score. In Eastern Mediterranean countries such as Iran, most of the injured motorcyclists are men [31, 32] and studies in the country have reported high men/women ratio in motorcycle accidents (16.9:1) [33] and (28:1) [34]. However, in other types of road accidents, as cited in a study conducted in Australia, about 40% of the injured participants were women [17]. One cohort study in Australia showed that being older and less hopeful to return to the job were reversely related to the physical health-related QOL score [17]. It is recommended that cohort studies be conducted with a few years of follow-up after the accidents for the motorcyclists in low- and middle-income countries (LMICs) to assess the QOL score. 

Concerning the clinical variables, the present study showed that injury to pelvis and knee reduced the QOL (had high QOL score), which is in line with the results of studies conducted in Canada and France [28, 30]. In the present study, no significant relationship was found between the length of hospitalization and QOL score of the injured people. The results of studies in Thailand [10] and Australia [12], however, demonstrated staying for one or more nights on hospital had a negative impact on the QOL score of the injured patient, which contradicts the results of the present study. Differences in the target group may be one of the reasons for this inconsistency. In an Australian study, patients who were hospitalized for more than seven days and those with severe injuries were excluded. Nevertheless, in the present study, about 15% of the injured were hospitalized for more than seven days and all injuries were included in the study. The hospitalization duration of less than half of the injured participants was not accurately documented.

In the present investigation, accidents that occurred on rainy days were reduced the QOL (had high QOL score), but those who were an accident with a vehicle had a better QOL (low QOL score) than others. According to the results of the previous studies conducted in India, the severity of accidents on rainy days is higher than that on sunny days [35]. Moreover, compared to car accidents, the severity of injuries from motorcycle accidents is higher. It seems, as expected, the severity of the accident could reduce the QOL.

The negative consequences of road accidents may be reduced by first aid, specialist transport, and emergency treatment of the victims [36]. Therefore, in low-and middle-income countries such as Iran, strengthening first aid courses have an accident notification system, and improving the co-operation of rescue services can be sufficient to improve post-accident medical care [37]. In these countries, interventions should not only be restricted to pre-accident or hospital, but also, after hospital discharge; countries should have serious plans, especially for high-risk groups. Extensive follow-up of patients and victims with low QOL should be considered after being discharged from the hospital. More specifically, it seems necessary to enhance and extend care, social support, psychological support, financial support, and rehabilitation for those at high risk of not fully recovering, such as very seriously injured victims as well as those with lower limb injuries such as injury to pelvis and knee. Moreover, early psychological monitoring and counseling are essential for the patients who experience even minor traffic accidents [38]. Therefore, successful interventions such as cognitive behavior therapy interventions should be used for patients in emergency departments and post-discharge by healthcare staff. Early identification and subsequent referral to treatment for depression as well as anxiety may decrease the likelihood of these conditions affecting long-term QOL [19, 38].

Assessing three-month post-accident showed that the QOL of the motorcyclists was improved. Since motorcycles are affordable vehicles for people in low- and middle-income countries and as reported by WHO, more than half of all road traffic fatalities involved motorcyclists, we expect that the results of this study would be a starting point for policy-makers and authorities’ extra efforts to improve motorcyclists’ safety levels. The following measures are suggested: producing standard and affordable helmets, considering separate routes for motorcyclists on roads, and providing more medical and insurance support to motorcyclists than users of other vehicles since their accident pattern and disability are different. Moreover, it is suggested that by designing multi-year cohort studies, the QOL of motorcyclists will be assessed for a longer time to identify the effective factors that improve the QOL and take appropriate measures accordingly. Social support, psychological support, financial support, and rehabilitation should be considered to improve their QOL in hospital discharge victims (especially for those with lower limb injuries such as injury to pelvis and knee). 

The study has several limitations: first, our exclusion criteria, those were in coma or could not talk could reduce the generalizability of the finding. Second, the sample size of this study was low; this limitation could reduce the generalizability. Third, this study represented the QOL of severely injured people and did not assess that of outpatients. Fourth, twice following-up in cohort study** are not sufficient; but** this cohort study was one of the first cohort studies conducted in Iran to evaluate the QOL of injured motorcyclists one and three months after the accidents. 

## List of Abbreviations

RTIs

Road Traffic Injuries

IIRTIRS

Iranian Integrated Road Traffic Injury Registry System

PTC

Persian Traffic Cohort

ICD-10

Classification of Diseases-10th revision

EQ-5D-3L

EQ-5D-3L questionnaire

PHQ 

Patient Health Questionnaire

PTSD 

Post-Traumatic Stress Disorder

HIS

Health Information System

GEE 

Generalized Estimating Equation
